# *Campylobacter* Species Isolated From Wild Birds in Switzerland and Comparison to Isolates From Food and Human Origin

**DOI:** 10.1002/mbo3.70176

**Published:** 2025-12-01

**Authors:** Marc J. A. Stevens, Gina Nadeen Buvoli, Lucien Kelbert, Nicole Cernela, Roger Stephan

**Affiliations:** ^1^ Institute for Food Safety and Hygiene, Vetsuisse Faculty University of Zurich Zurich Switzerland

**Keywords:** Campylobacter, One Health, whole genome sequences, wild birds

## Abstract

*Campylobacter* species, a major cause of gastroenteritis, have been frequently isolated from wild birds. Here we determined the prevalence of *Campylobacter* in wild birds from Switzerland. *Campylobacter* isolates were then further characterized by whole genome sequencing. A total of 154 samples from 27 different wild bird species were analyzed and *Campylobacter* was detected in 23 samples (14.9%). Twenty‐one isolates were identified as *C. jejuni,* one as *C. coli* and one isolate likely belongs to a novel species. Whole genome analyses revealed that the strains were diverse, belonging to 17 different sequence types. Antimicrobial resistances of the *C. jejuni* strains included class D ß‐lactamase *bla*_OXA_ genes in all isolates, T86I mutations in GyrA conferring resistance to quinolones in 7 isolates, and *tet*(O) in 3 isolates. A comparison to 787 *Campylobacter* from various sources in Switzerland showed that strains spread between humans, poultry and wild birds. Moreover, plasmid analyses and genome comparison provided a strong indication of horizontal gene transfer between *Campylobacter* strains. Our results strongly support a One‐Health approach that includes wild animals to understand and control epidemiology of *Campylobacter*.

## Graphical abstract

*Campylobacter* strains (Blue) from wild birds cluster with strains from poultry and human (red), showing dissemination of strains from wild birds via food to human.

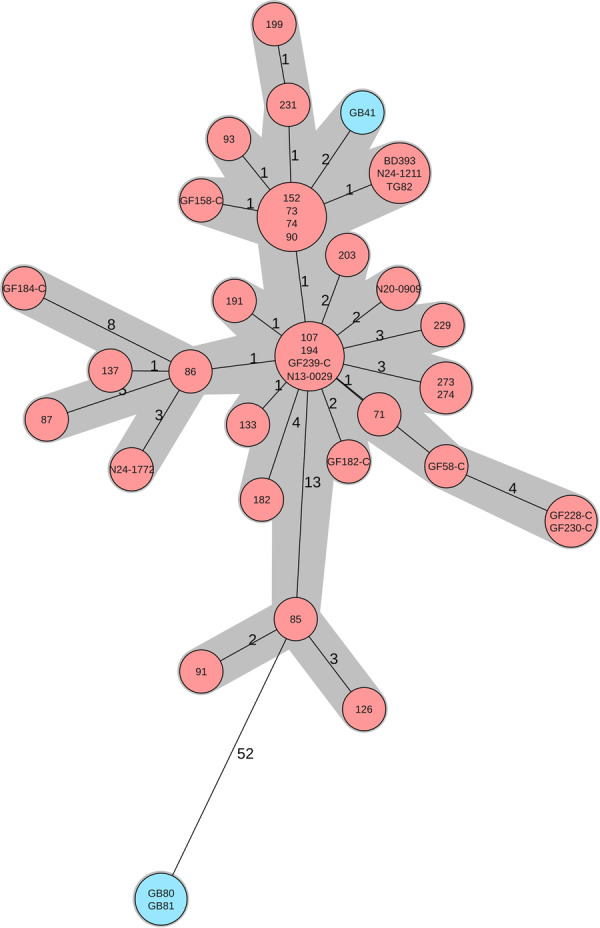

## 1 Introduction

*Campylobacter* species, particularly *Campylobacter jejuni* and *Campylobacter coli*, are significant zoonotic pathogens which cause gastroenteritis in humans (Chlebicz and Śliżewska 2018). Campylobacteriosis is a highly frequent disease with approximate 150.000 annual cases in the EU (EFSA EFSA, European Centre for Disease Prevention and Control ECDC 2023). Campylobacteriosis is mostly self‐limiting; however, severe complications such as the Guillain‐Barré syndrome or arthritis occur occasionally (Costa and Iraola 2019; Finsterer 2022). Moreover, a *Campylobacter* infection is associated with increased colorectal cancer risk (Kato et al. 2023) that is linked to production of the cytolethal distending toxin (He et al. 2024).

*Campylobacter* from poultry account for 50%–80% of campylobacteriosis cases in humans, followed by *Campylobacter* from cattle with 20%–30% (EFSA EFSA, European Centre for Disease Prevention and Control ECDC 2023). However, other animal such as wild birds are also sources of *Campylobacter *(Ahmed and Gulhan 2021). Wild birds account for approximately 2.5% of all *Campylobacter* infections in human (Lévesque et al. 2013; Mäesaar et al. 2020). In addition, *Campylobacter* from wild birds can be transmitted to poultry flocks and eventually to humans. Moreover, *Campylobacter* strains found in surface waters are also linked to wild birds due to fecal contamination (Mughini‐Gras et al. 2016).

Wild birds harbor various *Campylobacter* species, with *C. jejuni* generally being the most prevalent with 80%–90%, followed by *C. coli* and *C. lari* (Krawiec et al. 2017; Kwon et al. 2017; Mencía‐Gutiérrez et al. 2021). Recently, the novel species *C*. *molothri* was isolated from a variety of wild birds and from surface waters (Miller et al. 2025).

This cross‐sectional study investigated the prevalence of *Campylobacter* species in wild birds in Switzerland. Strains identified as *Campylobacter* were completely sequenced and compared to strains from human, slaughterhouse samples (neck skin samples of poultry) and retail poultry meat in Switzerland using core genome (cg) MLST.

## 2 Materials and Methods

### 2.1 Sample Collection

A total of 154 samples from various wild bird species were collected at three different Swiss wild bird rescue centers that is, Schweizerische Vogelwarte (Sempach, Switzerland), Stiftung Wildstation Landshut (Utzenstorf, Switzerland) and Greifvogelstation Berg am Irchel (Berg am Irchel, Switzerland) between October 2024 and January 2025. Birds were housed in individual cages, and samples taken regardless of sex, age, or reason for hospitalization. Fecal samples (*n* = 108) were collected from fresh feces using sterile cotton swabs. Rectum (*n* = 19) and intestine (*n* = 27) samples were collected from dead birds from the Swiss National Reference Centre for Poultry Diseases (Zurich, Switzerland).

Microbiological processing of samples was performed within 24 h of sampling. Samples were enriched in Preston Broth (Biolife Italiana, Milano, Italy) at 41.5°C for 24 h under microaerobic conditions. Subsequently, enrichment cultures were streaked onto Charcoal Cefoperazone Deoxycholateagar (CCDA; Oxoid Deutschland GmbH, Wesel, Germany) and incubated at 41.5°C under microaerobic conditions for 48 h.

### 2.2 Identification of Presumptive Positive *Campylobacter* Colonies

Single presumptive positive colonies were sub‐cultured on Columbia blood agar containing sheep blood (Thermo Fisher, Reinach, Switzerland) and identified using MALDI‐TOF‐MS (Flex Control 3.4, the MALDI Biotyper (MBT) Compass database version 4.1.100, and the MBT Compass BDAL 12.0 Library (Bruker, Zurich, Switzerland).

### 2.3 DNA Extraction and Whole Genome Sequencing

*Campyobacter* isolates were grown on Columbia blood agar containing sheep blood (Thermo Fisher, Reinach, Switzerland) at 42°C for 24 h under microaerobic conditions. Cryopreservation failed for three isolates, and these were therefore not sequenced. DNA isolation was performed using the DNA blood and tissue kit (Qiagen, Hombrechtikon, Switzerland) and DNA libraries were prepared using a Illumina DNA Prep(M) Tagmentation kit (Illumina, San Diego, CA, USA). Whole genome sequencing was performed on a MiniSeq sequencer (Illumina, San Diego, CA, USA) using a 2 × 150 reads strategy as described previously (Stevens et al. 2024a). Genomes were assembled using the skesa v2.5.1 based software Shovill 1. 1.0 (Seemann 2019) with default settings and a contig size cut off > 500 bp. The draft genomes were annotated using prokaryotic genome annotator prokka v1.14.6 (Seemann 2014a).

### 2.4 Bioinformatic Methodology

In general, all analyses were performed using so‐called default settings that is, the settings that are configured in a software application when first installed and are used by default when not altered.

Species were identified based on the average nucleotide identity (ANI) using a 94% identity as species cut off (Konstantinidis and Tiedje 2005). Genomes were compared to the 46 representative genomes of the genus *Campylobacter* downloaded from NCBI in March 2025, using fastANI v1.33 with default settings (Jain et al. 2018). Multi locus sequence typing (MLST) and core genome (cg) MLST analyses were performed in SeqSphere+ version 10.0.6 (Ridom GmbH, Münster, Germany) using the interspecies scheme for *C. jejuni* and *C. coli* from pubmlst. org (Jolley et al. 2018) and a CT threshold of thirteen alleles (Nennig et al. 2020). Minimal spanning trees were constructed in SeqSphere+ using default setting and with the “ignore empty values” option.

Antimicrobial resistance genes were identified using the Resistance Gene Identifier (RGI) 6.0.3 and database version 3.2.7 (Alcock et al. 2020 Jan), downloaded in March 2025. Point mutations that confer antibiotic resistances were identified using amrfinderplus 4.0.19 with the organism option “Campylobacter” activated (Feldgarden et al. 2021). Virulence genes were identified in a bidirectional‐best‐hit comparison of the proteomes against the core protein data set A from the virulence factor database (Liu et al. 2022). The comparison was performed using diamond v2.1.14 with an identity cut‐off of 70% (Buchfink et al. 2021).

Sequence alignments of CmeB were done using mafft v7.526 (Katoh 2002), with default settings. Trees were calculated using IQtree v2.0 (Minh et al. 2020), with the option “‐m MFP” for extended model selection followed by tree inference activated and using the clustal output from mafft as input. Midpoint‐rooted trees were visualized using Figtree v1.4.4 (Figtree Internet 2018).

Plasmid were identified with the mob_typer command from the Mob suite v3.1.9 (Robertson and Nash 2018) with default settings and draft genomes is input. Plasmids were classified according to types named by mob_typer. Distance of plasmids were calculated using the double cut join model in pling v2.0.0 using default setting (Frolova et al. 2024) and alignment were visualized using BRIGv0.95 (Alikhan et al. 2011).

Reactions were predicted using the KEGG orthologs (KOs) database. KOs were predicted by the online version of egg‐noggmapperv2 (Cantalapiedra et al. 2021) with default settings and the predicted proteomes as input. Proteomes were predicted by prokka v1.14.6 (Seemann 2014b) using default settings and the draft genomes as input.

**Figure 1:**
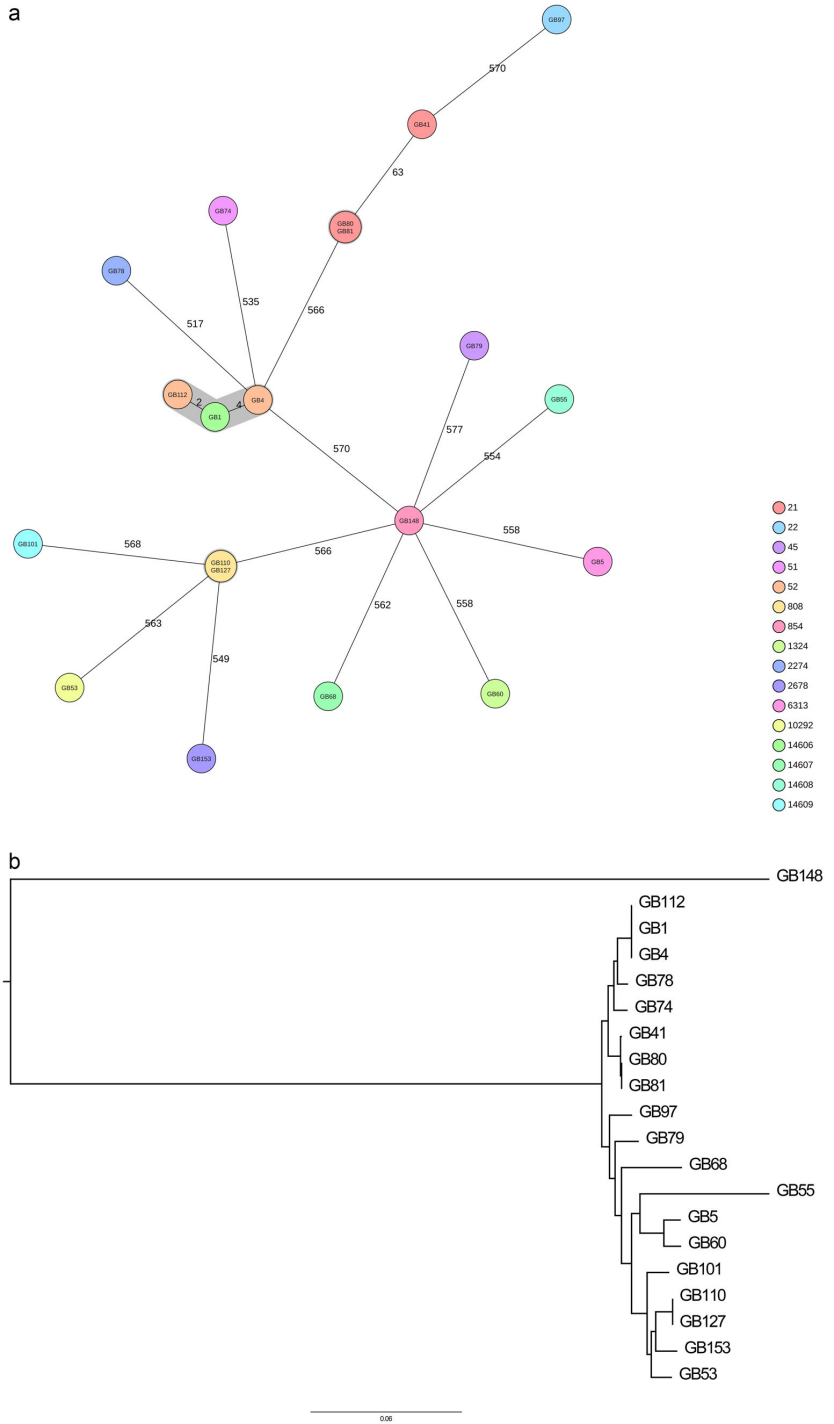
cgMLST‐based trees of 20 *Campylobacter* spp. isolated from wild birds in Switzerland. Isolated IDs and sequence types (ST) are indicated. (A) minimal spanning tree. (B) Neighborhood‐joining tree.

Horizontal gene transfer (HGT) was predicted as described by Inglin et al 2018 (Inglin et al. 2018). Shortly, the *C. jejuni* strains were divided into 2 subsets based on Figure 1B (Supporting Information S1: Table 4). Homologous proteins present between 1 and N minus 2 times in both sets were considered to be horizontally transferred.

## 3 Results

### 3.1 Prevalence of *Campylobacter* in Swiss Wild Birds

A total of 154 samples from 154 different birds and belonging to 27 wild bird species were taken (Supporting Information S1: Table 1). Samples derived from intestine samples (*n* = 27), rectum (*n* = 19) and from fresh feces (*n* = 108). Of the 154 samples, 23 (14.9%) were positive for *Campylobacter*. Seventeen of these 23 positive samples derived from feces (74%) and 6 from rectum (26%).

Of 23 positive samples, 9 derived from the common buzzard (*Buteo buteo*), corresponding to 12.3% of the common buzzard samples; 3 from the tawny owl (*Strix aluco*), corresponding to 25% of the samples; two from kestrel (*Falco tinnunculus*) and barn owl (*Tyto alba*), corresponding to 11.1% and 40% of the samples, respectively. Further, one positive sample derived from sparrowhawk (*Accipiter nisus*), sarrion crow (*Corvus corone*), blue tit (*Cyanistes caeruleus*), common Snipe (*Gallinago gallinago*), red kite (*Milvus milvus*), blackcap (*Sylvia atricapilla*), blackbird (*Turdus merula*), corresponding to between 20% and 100% of the samples (Supporting Information S1: Table 1).

### 3.2 Genomic Characteristics of *Campylobacter* Strains From Wild Birds

The genomes of 21 *Campylobacter* isolates from wild birds were sequenced using Illumina technology and the resulting draft genomes consisted of 11 to 123 contigs. The *Campylobacter* genomes sized between 1.6 and 2.1 Mbp bp. Eighteen isolates had ANIs *>* 96.4% to reference genome of *C. jejuni* NCTC 11168 (Accession NC_002163.1), classifying them as *C. jejuni*. Isolate GB55 had an ANI of only 94.7% to the reference strain, yet still above the 94% ANI cut off and hence GB55 was also classified as *C. jejuni*. Further, GB148 was classified as *C. coli* (Table 1). Isolate GB48 isolated from *Gallinago gallinago* (common snipe) had highest ANI to *C. lari*, but only at 92.4%. Hence GB48 could not be assigned to a sequenced *Campylobacter* species (Table 1).

**Table 1:** Strains presented in this study and their main characteristics.

Strain	ST	CT	Host	Sample	Genome accession	ANI^+^	Genome size	Contigs	N50	L50	Virulence factors
*Campylobacter jejuni*											
GB1	14606	440	*Tyto alba*	Fecal sample	JBMJBA000000000	98.40	1855498	72	181409	3	96
GB101	14609	8839	*Buteo buteo*	Instestine	JBMJAZ000000000	96.56	1816390	83	146168	3	85
GB110	808	8838	*Buteo buteo*	Instestine	JBMJAY000000000	96.68	1760898	59	119261	5	86
GB112	52	440	*Falco tinnunculus*	Fecal sample	JBMJAX000000000	98.31	1646937	13	1034049	1	99
GB127	808	8838	*Buteo buteo*	Fecal sample	JBMJAW000000000	96.74	1760882	60	104643	6	86
GB153	2678	8842	*Buteo buteo*	Instestine	JBMJAU000000000	96.81	1794224	35	151911	4	87
GB4	52	440	*Tyto alba*	Fecal sample	JBMJAT000000000	98.41	1633065	11	336610	2	97
GB41	21	2570	*Strix aluco*	Fecal sample	JBMJAS000000000	99.59	1704820	20	154547	3	121
GB5	6313	8833	*Sylvia atricapilla*	Fecal sample	JBMJAQ000000000	96.41	1728127	45	102179	5	90
GB53	10292	8834	*Corvus corone*	Fecal sample	JBMJAP000000000	96.62	1789592	84	115716	5	81
GB55	14608	8832	*Buteo buteo*	Fecal sample	JBMJAO000000000	94.77	1607911	24	139993	3	93
GB60	1324	8835	*Turdus merula*	Fecal sample	JBMJAN000000000	96.52	1614846	27	111935	4	93
GB68	14607	8831	*Cyanistes caeruleus*	Fecal sample	JBMJAM000000000	96.60	2088825	123	103871	7	86
GB74	51	8836	*Strix aluco*	Fecal sample	JBMJAL000000000	98.50	1671989	14	293733	2	92
GB78	2274	2445	*Strix aluco*	Fecal sample	JBMJAK000000000	98.43	1797532	31	248539	3	95
GB79	45	1688	*Buteo buteo*	Rectum	JBMJAJ000000000	97.61	1649234	19	205122	4	98
GB80	21	8837	*Falco tinnunculus*	Fecal sample	JBMJAI000000000	99.61	1654012	13	189475	3	122
GB81	21	8837	*Accipiter nisus*	Fecal sample	JBMJAH000000000	99.59	1653635	14	189475	3	124
GB97	22	8840	*Milvus milvus*	Instestine	JBMJAG000000000	97.90	1617815	22	184961	3	92
*Campylobacter coli*											
GB148	854	8841	*Buteo buteo*	Fecal sample	JBMJAV000000000	98.93	1695372	23	271767	3	84
*Campylobacter* spec.											
GB48	na	Na	*Gallinago gallinago*	Fecal sample	JBMJAR000000000	na	1670620	108	107371	5	29

Abbreviations: ANI = average nucleotide identity in percentage to C. jejuni or C. coli reference genome, CT = complex type, ST = sequence type.

### 3.3 Sequence Type of *Campylobacter* Isolates From Wild Birds and Clonal Relationship to Other Isolates

Next, core genome (cg) MLST was used to determine the relation between the isolates. Classical MLST is based on 7 genes, whereas cgMLST is based on 654 genes, resulting in a much higher resolution for strain comparison (Nennig et al. 2020). The isolate GB48 was omitted from cgMLST analysis because only 19.7% of the core genes could be identified in the isolate. The *Campylobacter* isolates from wild birds belong to 17 different sequence types (STs; Figure 1a), showing they are diverse. ST21 is represented 3 times, and ST52 and ST808 twice. ST21 isolates GB80 and GB81 are clonal. Both isolates were from samples from Landshut taken on the same day; one sample from *Falco tinnunculus* (common kestrel) and one from *Accipiter nisus* (sparrowhawk). The ST808 isolates GB110 and GB127 are also clonal, both isolated from *Buteo buteo* (common buzzard) in Berg am Irchel. Further, the isolates GB1, GB4, and GB112 are clonal and assigned to complex type (CT) 440 (Figure 1a). Remarkably, isolate GB4 and GB122 belong to ST52, whereas GB1 belongs to ST14606 (Figure 1a). All three isolates were from the “wildstation Landshut”, two from a Barn owl, a one from a Common buzzard (Table 1).

To obtain a more comprehensive overview of the genetic relations of the isolates, we constructed a neighborhood‐joining tree based on the cgMLST distances (Figure 1B). *C. coli* strain GB148 is an outlier, as expected. One branch contains 8 strains and these strains seem closely related, the other branch contains 11 strains and the strains are more diverse.

**Figure 2:**
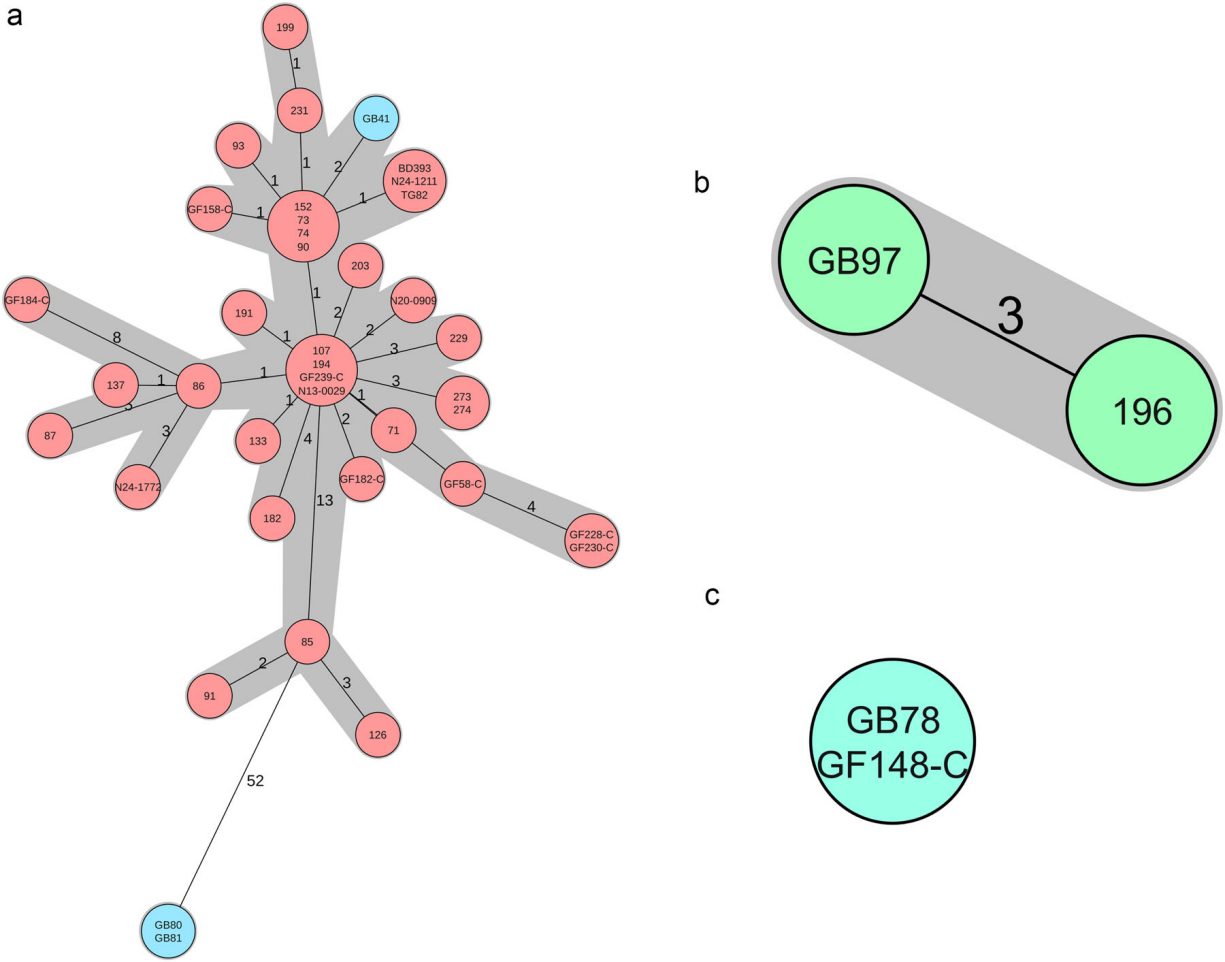
Clonality of wild bird isolates with *Campylobacter* isolates from other sources in Switzerland. (a) ST21, CT2570. Strains from birds are labeled blue; (b) ST22, CT8840; (c) ST2274, CT1688.

The sequences of 787 *Campylobacter* isolates (2009 to 2025) from various sources in Switzerland were available for further comparison (Ghielmetti et al. 2023; Stevens et al. 2024b; Kelbert et al. 2025). The ST21 CT2570 isolate GB41 clustered in a cgMLST analysis with 37 isolates deriving from human (*n* = 6), neck skin samples from poultry of a slaughterhouse (*n* = 24) and poultry meat collected at retail level (*n* = 6, Figure 2a). Further, isolate GB97 belonging to ST22 clustered with the neck skin isolate 196 from slaughterhouse (Stevens et al. 2024b) and isolate GB78 belonging to ST2274 clustered to isolate GF148‐C (poultry raw meat at retail level; (Kelbert et al. 2025)). The occurrence of the same clone in poultry and wild birds, shows that transfer of strains between wild birds and poultry production occurs.

### 3.4 Antimicrobial Resistance Genotypes of *Campylobacter* Isolated From Wild Birds

In a next step, the antimicrobial resistance (AMR) gene profiles of the sequenced isolates were determined. Eighteen of the twenty *Campylobacter* isolates harbored the multidrug efflux‐pump operon *cmeABC* that is regulated by *cmeR* (Table 2). Only GB48 and GB148 did not possess a *cmeABC* operon. Recently, a resistance‐enhancing (RE) CmeB variant (Yao et al. 2016) was identified in Swiss strains (Kelbert et al. 2025). Isolate GB55 has a CmeB that clusters distinct from the CmeBs of the other wild bird strain but closely to the RE‐variant NT161 (Supporting Information S1: Figure 1). Moreover, CmeB of this isolate GB55 has only 5 AA changes compared to NT161, strongly suggesting that GB55 harbors a RE *cmeABC* operon.

**Table 2:** Antimicrobial resistance genes and point mutations that confer antibiotic resistances in *Campylobacter* strains from wild birds.

Strain	*gyrA* a	*cmeA*	*cmeB*	*cmeC*	*cmeR*	*tet*(O)	OXA‐type
GB1	Yes	Yes	Yes	Yes	Yes	—	OXA‐603
GB101	—	Yes	Yes	Yes	Yes	—	OXA‐637
GB110	—	Yes	Yes	Yes	Yes	—	OXA‐446
GB112	Yes	Yes	Yes	Yes	Yes	—	OXA‐603
GB127	—	Yes	Yes	Yes	Yes	—	OXA‐446
GB148	Yes	—	Yes	—	—	Yes	OXA‐193
GB153	—	Yes	Yes	Yes	Yes	—	OXA‐446
GB4	Yes	Yes	Yes	Yes	Yes	—	OXA‐603
GB41	Yes	Yes	Yes	Yes	Yes	—	OXA‐193
GB5	—	Yes	Yes	Yes	Yes	—	OXA‐617
GB53	—	Yes	Yes	Yes	Yes	—	OXA‐614
GB55	—	Yes	Yes	Yes	Yes	—	OXA‐446
GB60	—	Yes	Yes	Yes	Yes	—	OXA‐447
GB68	—	Yes	Yes	Yes	Yes	—	OXA‐465
GB74	Yes	Yes	Yes	Yes	Yes	Yes	OXA‐184
GB78	Yes	Yes	Yes	Yes	Yes	Yes	OXA‐193
GB79	—	Yes	Yes	Yes	Yes	—	OXA‐193
GB80	—	Yes	Yes	Yes	Yes	—	OXA‐193
GB81	—	Yes	Yes	Yes	Yes	—	OXA‐193
GB97	—	Yes	Yes	Yes	Yes	—	OXA‐193

a T86I mutation in *gyrA* resulting in resistance to quinolones.

The *C. jejuni* and *C. coli* isolates harbored a class D ß‐lactamase *bla*_OXA_ gene, which belonged to one of 9 different variants (Table 2). Further, 7 isolates had a GyrA T81I mutation conferring quinolone resistance and 3 strains harbored the ribosome protection protein gene *tet*(O) conferring tetracycline resistance (Table 2). The isolates that harbored *tet*(O), i.e. GB74, GB78, and GB148, also had the GyrA (T81I) mutation, making them MDR strains harboring *bla*_OXA_‐*gyrA* (T86I)‐*tet*(O).

AMR genes such as *aad* (He et al. 2024), *aph(3′)‐IIIa*, *sat‐4*, previously detected in Swiss *Camplyobacter* strains (Ghielmetti et al. 2023; Kelbert et al. 2025; Stevens et al. 2025), were not found in wild bird strain Further, the unclassified strain GB48 did not harbor any AMR genes.

### 3.5 Distribution of Virulence Determinants Among *Campylobacter* Isolated From Wild Birds

The wild bird *C. jejuni* isolates harbored between 80 and 123 predicted virulence factors (Table 1). All but one VFs are typical *Campylobacter* VFs, the only exception being a S‐ribosylhomocysteinase originating from *Vibrio cholerae*, present in 13 isolates (Supporting Information S1: Table 2). Sixty‐eight VFs were found in all isolates and are mainly involved in flagella, colonization, and capsular polysaccharides (CPS). VFs that encode sialylated lipooligosaccharides (LOLs) were CstIII (*n* = 4), NeuA1 (*n* = 3), NeuB1 (*n* = 8), NeuC1 (*n* = 5), and WlaN (*n* = 1). The combination CstIII and NeuABC was found in isolate GB41 and the clonal isolates GB80 and GB81. The WlaN was only detected in GB55.

The cytolethal distending toxins CdtA, CdtB, and CdtC were found in 10, 19, and 12 isolates, respectively.

*C. coli* GB148 harbored 84 VFs and the nonclassified strain GB48 harbored 29 VFs (Table 1).

### 3.6 Mobile Genetic Elements and Potential of Horizontal Gene Transfer

The 21 *Campylobacter* isolates were predicted to harbor between 1 and 5 plasmids (Table 3). In total, 35 plasmids were found which belonged to 10 different types. The plasmid type pAG887 was present in 19 isolates, pAE190 in 7 isolates, pAC320 in 3 isolates and pAD894 in 2 isolates. Other plasmids were only present in single isolate. None of the plasmid harbored AMR genes.

**Table 3:** Plasmids identified in this study.^a^

Strain	pAB469	pAC319	pAC320	pAC321	pAC502	pAD894	pAE190	pAF478	pAG887	pnovel
GB1	0	0	0	0	0	0	0	0	1	0
GB101	0	0	0	0	0	0	1	0	1	0
GB110	0	0	0	0	0	0	1	0	1	0
GB112	0	0	0	0	0	0	0	0	1	0
GB127	0	0	0	0	0	0	1	0	1	0
GB148	0	0	0	0	0	1	0	0	0	0
GB153	0	0	0	0	1	0	1	0	1	0
GB4	0	0	0	0	0	0	0	0	1	0
GB41	0	0	0	0	0	0	0	0	1	0
GB48	0	1	0	0	0	0	0	0	0	0
GB5	0	0	0	0	0	0	0	0	1	1
GB53	0	0	0	0	0	1	1	0	1	0
GB55	0	0	0	0	0	0	0	0	1	0
GB60	0	0	0	0	0	0	0	0	1	0
GB68	1	0	0	1	0	0	1	1	1	0
GB74	0	0	1	0	0	0	0	0	1	0
GB78	0	0	0	0	0	0	1	0	1	0
GB79	0	0	0	0	0	0	0	0	1	0
GB80	0	0	1	0	0	0	0	0	1	0
GB81	0	0	1	0	0	0	0	0	1	0
GB97	0	0	0	0	0	0	0	0	1	0
**Total**	*1*	*1*	*3*	*1*	*1*	*2*	*7*	*1*	*19*	*1*

a 0 = absent; 1 is present.

Comparison of the predicted plasmids in wild bird isolates to the predicted plasmids from the 787 Swiss *C. jejuni* and *C. coli* isolates, revealed that plasmids pAC319 and pAF478 were only present in wild bird isolates. The predicted 52.6‐kbp pAC319 was found in unclassified strain GB48 and encodes the Cag pathogen island protein 12 and VirB Type IV secretion system proteins (Supporting Information S1: Table 3), suggesting a role in virulence. Plasmid pAF478 is predicted to be 91.4 kbp and encodes only hypothetical genes or genes involved in DNA modification.

Plasmid pAG887 was present in 92% of the 787 sequenced Swiss strains and in 95% of wild bird isolates, showing that is it almost ubiquitous in Swiss *Campylobacter* (Table 3). The plasmids seem to play a role in virulence, because between 6 and 10 VFs are located on them. These are proteins involved in flagellum biosynthesis and in some strains also cytolethal distending toxin A and C (data not shown).

**Figure 3:**
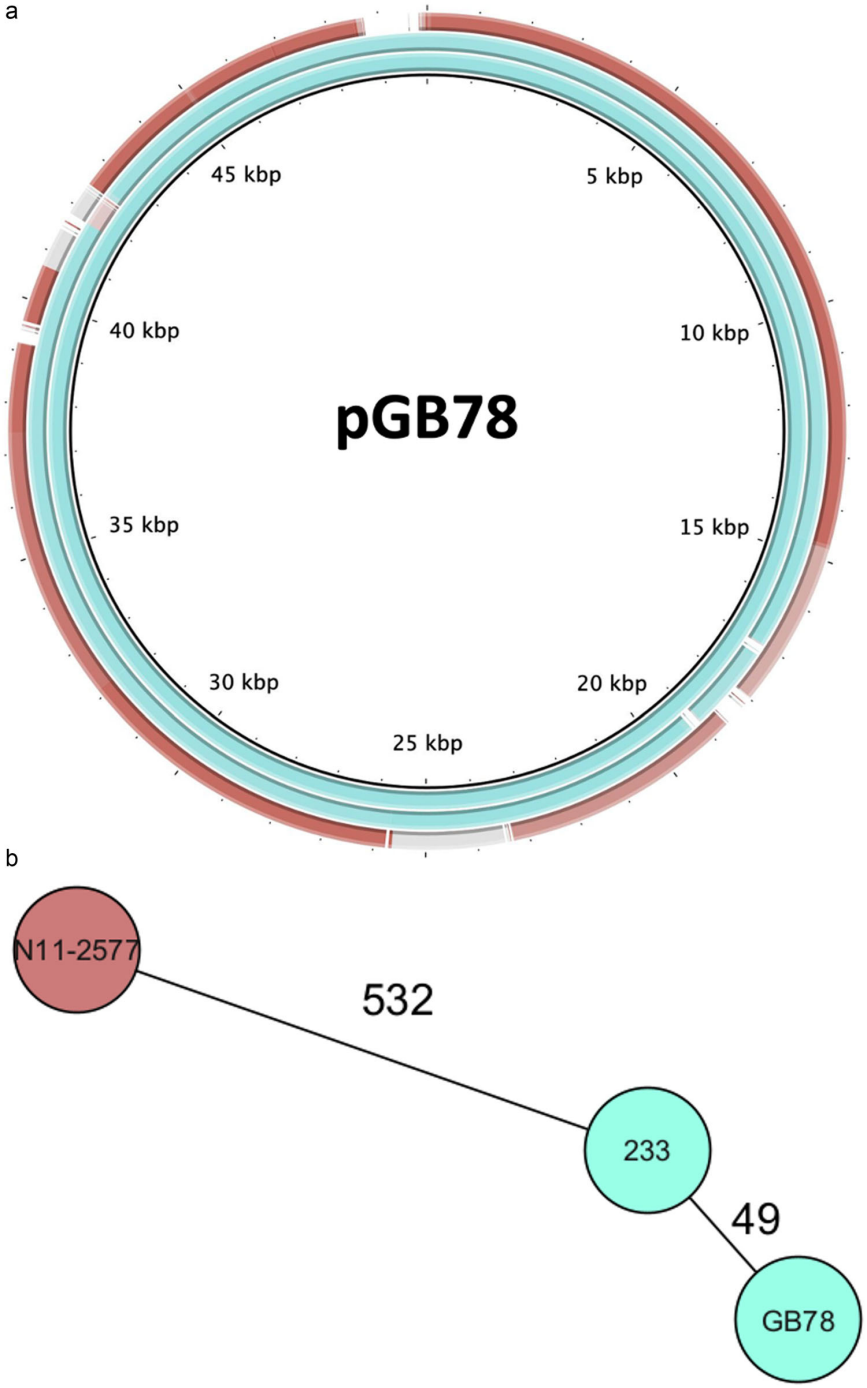
Horizontal gene transfer involving *Campylobacter* isolates from wild birds. (a) The plasmids p233_AE190 (blue) and pN11‐2577_AE190 (red) plotted on pGB78_AE190 (blue). Colored region share a homology of > 99.5% identity, grayish regions of 95%. (b) Strains 233 (blue), N11‐2577 (red) and GB78 (blue) in a cgMLST comparison.

Plasmid type pAE190 was present 33 of the 787 strains, corresponding to 4.3%. The plasmids belonging to this type are diverse, exemplified by the fact that the core genome of all pAE190 plasmids consists of only a single gene (data not shown). However, some of the pAE190 plasmids are virtually identical, with a Double Cut and Join model based distance < 10. This is exemplified by pAE190 from GB78 and 233, which share two 15‐kbp regions > 99.5% identity with pAE190 from N11‐2577, a human isolate (Figure 3a). This, although the ANI between strain GB78 and N11‐2577 is < 98.7% and they are unrelated in the cgMLST comparison (Figure 3b). This suggest that HGT occurred between strain from wild bird and other origins. The function encoded on pAE190 are hypothetical or involved in DNA recombination, which is in line with the observation that it is a rapidly changing plasmid.

In conclusion, the plasmid distribution in *Camplybacter* strains from variate Swiss sources, strongly suggests that besides strain transfer, also gene transfer occurs between isolates from wild birds, poultry and human.

An attempt to identify additional mobile genetic elements (MGEs) using the mobile elements finder (Johansson et al. 2021), revealed only 3 strains carrying an insertion (data not shown), which encoded a transpose and hypothetical functions. These insertions were not further explored.

Plasmids and other MGE are involved in HGT and we searched for proof of HGT between our strains. A strong indication for HGT is the unique presence of homologous proteins in a subset of strains that are closely related. Therefore, we searched for proteins that are homologous and found in a subset of strains in each of the two branches in the neightborhood‐joining tree (Figure 1b). In total, 219 proteins were identified that were predicted to be horizontally transfered between *C. Jejuni* strains of which 177 had a hypothetical functions (Supporting Information Table 4).

The occurrence of similar plasmids in little related strains and the identification of 219 proteins that were potentially transferred between *C. Jejuni* strains, strongly point towards HGT.

### 3.7 Biosynthetic Genes Cluster in *Campylobacter* Strains From Swiss Wild Birds

A total of 1293 functional KEGG ortholog reactions were predicted to be present in our strains; between 1031 and 1099 in the *C. jejuni* strains, 1069 in *C. coli* GB148 and 1023 in *C*. spec GB48 (Supporting Information S1: Table 5). The *C. jejuni* core‐reactome, i.e. reactions found in all strains, consisted of 983 reactions. The pan‐reactome, i.e. the collection of all reactions, consisted of 1237 reactions, meaning that 254 reactions are not ubiquitous in *C. jejuni*. Reactions needed for biosynthesesis of compounds for which *C. jejuni* is generally prototroph, seemed to be part of the core‐reactome. The only exception is that a tryptophan synthase is missing in GB68, suggesting that the strain is auxotroph for tryptophan. More interesting are functions which give a potential advantage in a certain environment. Strain GB1 has additional mannose/fructose transporter function, sugars on which *C. jejuni* is not able to grow. Further, ImpLJHGCB proteins are involved in bioflim formation, and found in 6 strains. Other proteins such as Vir proteins, which are involved in flagella formation, or cag pathogenicity island proteins were found in 4 strains.

## 4 Discussion

In this study, samples from wild birds were collected and analyzed for the presence of *Campylobacter*. The prevalence of *Campylobacter* in Swiss birds was 14.9%. Several studies explored the prevalence of *Campylobacter* in wild birds: An Italian study focusing on birds of prey found a prevalence of 33.1% for *C. jejuni* whereas a Spanish study found a prevalence of 5.9% in decoy birds for hunting and of 22.7% several raptor species (Gargiulo et al. 2018; Jurado‐Tarifa et al. 2016). The prevalence in northern England seems much lower, only 1.4% of wild birds samples were positive for *Campylobacter* spp (Hughes et al. 2009). A non‐culture based study revealed approximate 10% prevalence in European birds (Poosakkannu et al. 2025). Variations in *Campylobacter* prevalence in wild birds are due to different locations, seasons, species, sample types, methodology, ecological factors and health status of wild birds (Ahmed and Gulhan 2021; Ishihara et al. 2012). In our study, we analyzed samples from sick or injured birds in winter, which likely affected the prevalence of *Campylobacter*. Nevertheless, the prevalence was in the range of those found in other European studies.

Twenty *C. jejuni* isolates, one *C. coli*, and one unknown species were isolated from wild birds and completely sequenced. The population was diverse with 17 different STs, which parallels observations in other Swiss environments (Stevens et al. 2024b; Kelbert et al. 2025). The diversity of *Campylobacter* in Swiss wild birds is also reflected by strain GB48 that seems to belong to a novel species, although tests beyond an ANI analysis should be performed. Strain GB48 was isolated from a common snipe, the only migratory bird that was positive for *Campylobacter* in our study. Migratory birds can transmit *Campylobacter* species to poultry (Tawakol et al. 2023). Another novel *Campylobacter* species, i.e. *C. molothir*, has been isolated from wild birds recently (Miller et al. 2025) and wild birds might be a source for more undiscovered *Campylobacter* species and variants.

Seven out of 22 wild‐bird isolates had the GyrA (T81I) mutation conferring resistance to quinolones. This is a lower rate compared to *C*. *jejuni* strains from poultry, neck skin samples at slaughterhouses and humans isolated in Switzerland (Ghielmetti et al. 2023; Kelbert et al. 2025; Stevens et al. 2025). AMR genes such as *aad* (He et al. 2024 *)*, *aph(3′)‐IIIa*, *sat‐*4, previously found in Swiss strains (Ghielmetti et al. 2023; Kelbert et al. 2025; Stevens et al. 2025), bit were absent in our isolates. Hence, the resistome of *C*. *jejuni* from wild birds is smaller compared to that of other environments. Nevertheless, the occurrence of a resistance‐enhancing variant RE‐cme*ABC* operon is a risk for spread of this variant, because wild birds are a reservoir for antimicrobial‐resistant *Campylobacter* (Hock et al. 2024).

Virulence factors included the cytolethal distending toxin operon *cdt*ABC, which is involved in colorectal cancer development (Kato et al. 2023; He et al. 2024), and lipooligosaccharides (LOLs) which are associated with the Guillain–Barré syndrome (Finsterer 2022). Only one isolate, GB55, carried the LOL WlaN. This had an ANI of only 94.7% to the reference strain and is the only strain that carries an RE CmeABC transporter. This suggests that wild birds might be a source for novel *C*. *jejuni* variants and that such variants can bring novel genes into the gene pool of *C*. *jejuni* linked to human or poultry.

The plasmid type pAG887 is found in > 90% of the Swiss strains. Its size > 90 kbp and the fact that it harbors several virulence factors, suggests that it is a pVIR plasmid. However, Campylobacter pVIR plasmids carry type IV secretion systems (Bacon et al. 2000; Y. He et al. 2025), whereas pAG887 carries flagella genes. Nevertheless, pAG887 like plasmids are a concern as some of them carry cytolethal distending toxins A and C, which are involved in colorectal cancer development (He et al. 2024). Plasmid type pAE190 did not harbor factor involved in virulence or AMR, but it spread suggests a role in HGT.

HGT can also explain the presence of fructose/mannose transporter functions and functions involved biofilm formation in a subset of our strains (Supporting Information Table 5). Such acquired functions will give strains a benefit in certain environments. Over 200 proteins were found that were potentially transferred horizontally, which is in line with the ability of *C. jejuni* to be naturally competent (Bae et al. 2014).

We found 3 isolates from wild birds which were clonal to isolates from poultry, and one isolate clonal to a human strain. The occurrence of these clones suggests that *C. jejuni* from wild birds belong at least partially to the same population as *C. jejuni* in poultry and, via poultry, in human. Therefore, epidemiological studies of *Campylobacter* infection should not ignore wild birds (Ahmed and Gulhan 2021). Wild birds, especially migratory, may even introduce new variants and genes into the population, especially migratory bird (Tawakol et al. 2023), like the unknown species GB48 and the RE‐*cme*ABC carrying, atypical *C. jejuni* GB55 described in this study. A one‐health approach including wild animals is therefore needed to understand the spread, epidemiology and evolution of *Campylobacter*.

## Author Contributions

**Marc J. A. Stevens:** conceptualization (supporting), writing – original draft (lead), formal analysis. **Gina Nadeen Buvoli:** formal analysis, writing – review and editing. **Lucien Kelbert:** conceptualization (supporting), formal analysis, writing – review and editing. **Nicole Cernela:** formal analysis, writing – review and editing. **Roger Stephan:** conceptualization (Lead), writing – original draft (supporting), writing – review and editing.

## Ethics Statement

Ethical approval was not required, since no samples were taken directly from living birds. Biological material can be obtained from the Institute for Food Safety and Hygiene.

## Conflicts of Interest

The authors declare no conflicts of interest.

## Supporting information


**Supporting Figure 1:** Neighbor‐joining tree of the aligned amino acid sequence of CmeB from the 19 *C. jejuni* strains presented in this study. CmeB from *C. jejuni* NT161 (GenBank accession number KT778507.1) is included as a reference for the resistance‐enhancing (RE) CmeB variant (red branch).

**Supporting Table 1:** Table with all information on the biosamples presented in this study.

**Supporting Table 2:** Table with virulence factors in the genomes presented in this study.

**Supporting Table 3:** List of encoded functions on the plasmids predicted in the isolates from this study.

**Supporting Table 4:** Proteins found to be transferred horizontal between strains. Yellow cells: presence matrix strains from branch 1; Blue cells: presence matrix strains from branch 2.

**Supporting Table 5:** Presence‐absence matrix of KEGG orthologs in the 21 strains.

## Data Availability

Sequencing read data and genome assemblies have been deposited under BioProject accession number PRJNA1223158. The Whole Genome Shotgun projects have been deposited at DDBJ/ENA/GenBank under the accession numbers JBMJAG000000000 to JBMJBA000000000.
