# Supplementary figures and images for: Campylobacter Species Isolated From Wild Birds in Switzerland and Comparison to Isolates From Food and Human Origin
Source: Microbiologyopen. 2025 Dec 1;14(6):e70176. doi: 10.1002/mbo3.70176 (PMC12666463; doi:10.1002/mbo3.70176)

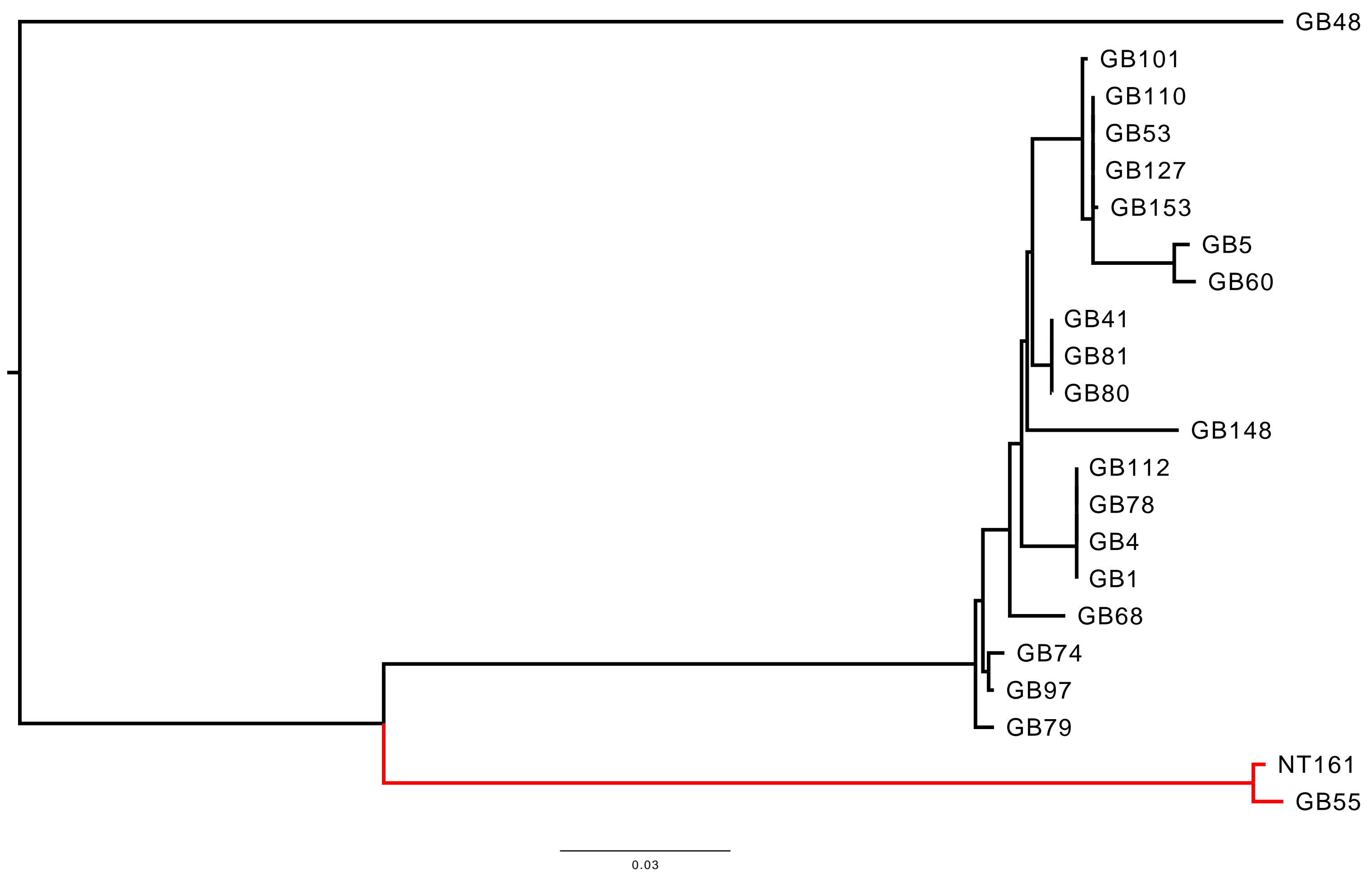

Supplement: Supplementary file 1 — Supporting Figure 1: Neighbor‐joining tree of the aligned amino acid sequence of CmeB from the 19 C. jejuni strains presented in this study. CmeB from C. jejuni NT161 (GenBank accession number KT778507.1) is included as a reference for the resistance‐enhancing (RE) CmeB variant (red branch). [file MBO3-14-e70176-s003.pdf]
